# Community-acquired Pneumonia in People With HIV During the Current Era of Effective Antiretroviral Therapy: A Multicenter Retrospective Cohort Study

**DOI:** 10.1093/cid/ciae393

**Published:** 2024-07-27

**Authors:** Anthony D Bai, Siddhartha Srivastava, Jorge L Martinez Cajas, Fahad Razak, Amol A Verma

**Affiliations:** Division of Infectious Diseases, Department of Medicine, Queen's University, Kingston, Ontario, Canada; Division of General Internal Medicine, Department of Medicine, Queen's University, Kingston, Ontario, Canada; Division of Infectious Diseases, Department of Medicine, Queen's University, Kingston, Ontario, Canada; Department of Medicine, University of Toronto, Toronto, Ontario, Canada; Li Ka Shing Knowledge Institute, St. Michael's Hospital, Unity Health Toronto, Toronto, Ontario, Canada; Institute of Health Policy, Management and Evaluation, University of Toronto, Toronto, Ontario, Canada; Department of Medicine, University of Toronto, Toronto, Ontario, Canada; Li Ka Shing Knowledge Institute, St. Michael's Hospital, Unity Health Toronto, Toronto, Ontario, Canada; Institute of Health Policy, Management and Evaluation, University of Toronto, Toronto, Ontario, Canada

**Keywords:** human immunodeficiency virus (HIV), AIDS, community-acquired pneumonia, mortality, cohort study

## Abstract

**Background:**

It is unclear if human immunodeficiency virus (HIV) affects the prognosis for community-acquired pneumonia (CAP) in the current era of effective antiretroviral therapy. In this multicenter retrospective cohort study of patients admitted for CAP, we compared the in-hospital mortality rate between people with HIV (PWH) and those without.

**Methods:**

The study included consecutive patients admitted with a diagnosis of CAP across 31 hospitals in Ontario, Canada, from 2015 to 2022. HIV infection was based on discharge diagnoses and antiretroviral prescription. The primary outcome was in-hospital mortality. Competing risk models were used to describe time to death in hospital or discharge. Potential confounders were balanced using overlap weighting of propensity scores.

**Results:**

Of 82 822 patients admitted with CAP, 1518 (1.8%) had a diagnosis of HIV. PWH were more likely to be younger, male, and have fewer comorbidities. In the hospital, 67 (4.4%) PWH and 6873 (8.5%) people without HIV died. HIV status had an adjusted subdistribution hazard ratio of 1.02 (95% confidence interval, .80–1.31; *P* = .8440) for dying in the hospital. Of 1518 PWH, 440 (29.0%) patients had a diagnosis of AIDS. AIDS diagnosis had an adjusted subdistribution hazard ratio of 3.04 (95% CI, 1.69–5.45; *P* = .0002) for dying in the hospital compared to HIV without AIDS.

**Conclusions:**

People with and without HIV admitted for CAP had a similar in-hospital mortality rate. For PWH, AIDS significantly increased the mortality risk. HIV infection by itself without AIDS should not be considered a poor prognostic factor for CAP.

## Teaser

In this large multicenter retrospective cohort study, HIV status by itself was not a risk factor for in-hospital mortality in community-acquired pneumonia. People with HIV who did not have AIDS had a significantly lower mortality risk than people with AIDS.

## Graphical Abstract

**Graphical Abstract:**
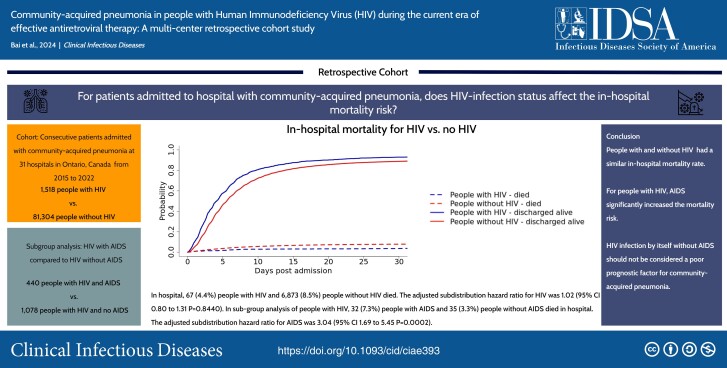
This graphical abstract is also available at Tidbit: https://tidbitapp.io/tidbits/community-acquired-pneumonia-in-people-with-human-immunodeficiency-virus-hiv-during-the-current-era-of-effective-antiretroviral-therapy-a-multi-center-retrospective-cohort-study?utm_campaign=tidbitlinkshare&utm_source=IO

Community-acquired pneumonia (CAP) is a common infection that is treated in hospital. The annual incidence is estimated to be 650 per 100 000 in the general population and the 1-month mortality is estimated to be between 5% to 10% [1–3].

In people with human immunodeficiency virus (HIV), CAP incidence has been decreasing likely due to improved care, effective HIV treatment and decreasing proportion of smokers [4]. The incidence of CAP in people with HIV (PWH) and people without HIV are now likely similar [4].

Historically, PWH with CAP had a higher risk of complication [5] and mortality [6–9]. In theory, if HIV is virologically suppressed with a normal CD4 T-lymphocyte count in the current era of early and effective antiretroviral therapy (ART), PWH should have a normal immune system and thus have the same prognosis as people without HIV. More recent studies that compared the prognosis between PWH and people without HIV focused on CAP subgroups such as CAP resulting from *Streptococcus pneumoniae* [10] and CAP in older male veterans [11]. Therefore, the prognosis of PWH hospitalized with CAP in general is still unclear in the current era of ART.

It is important to describe PWH hospitalized for CAP because the current CAP guidelines do not address this patient population [12]. As a result, some clinicians may continue to view PWH with CAP as a high-risk population, resulting in broader antibiotic coverage, longer antibiotic duration, and longer hospital stay.

We conducted a large multicenter retrospective cohort study of patients admitted to the hospital with a diagnosis of CAP to compare in-hospital mortality between PWH and people without HIV.

## METHODS

This was a multicenter retrospective cohort study across 31 hospitals in Ontario, Canada (Supplementary Table 1). The Unity Health Toronto research ethics board approved this study (20–2163-344). The study was reported per the Strengthening the Reporting of Observational Studies in Epidemiology guidelines [13] (Supplementary Table 2).

### Data Source

The GEMINI database included internal medicine and intensive care unit (ICU) patients admitted to participating hospitals in Ontario, Canada [14, 15]. This database included administrative and clinical data linked at the individual patient level for the patient's entire hospital stay from the initial emergency room visit to discharge as well as readmission. Data included patient demographics, diagnoses, interventions, resource use, medications, bloodwork, and disposition. Diagnoses were classified using the International and Statistical Classification of Diseases and Related Health Problems, Tenth Revision, Canada (ICD-10-CA) [14, 15].

### Patient Population

This study included consecutive patients admitted to a medical inpatient service or ICU at 31 acute care hospitals in Ontario, Canada, from 1 January 2015 to 1 July 2022 with a diagnosis of CAP based on ICD-10-CA codes of J10 to J18 as the most responsible diagnosis on the hospital discharge summary [16, 17]. In addition, HIV diagnosis or chronic obstructive pulmonary disease as the most responsible admitting diagnosis with additional ICD-10-CA codes of J10 to J18 were also included, because coding convention necessitated listing those conditions as primary when they coexisted with CAP. This approach had been previously validated to accurately capture pneumonia cases with sensitivity and specificity of 98% and 97% respectively [18]. The study dates determined the sample size.

Patients admitted for pulmonary tuberculosis or *Pneumocystis jirovecii* were not included in the study based on the above definition because these infections were distinct from CAP in terms of risk factors, clinical presentation, treatment, and prognosis.

### Exposure Status

HIV infection status was determined based on ICD-10-CA codes related to HIV and AIDS (Supplementary Table 3). The diagnoses were further classified into HIV with AIDS and HIV without AIDS based on ICD-10-CA codes for AIDS or AIDS defining illnesses [19]. AIDS diagnosis was based on the current hospital admission. To corroborate HIV infection status, we also searched for the same HIV ICD-10-CA codes within patient's prior admission records and presence of any HIV-specific ART medication at any time during the current hospital stay (Supplementary Table 4). Patients were still categorized as PWH if they had a diagnosis of HIV in current hospital admission without prior history and not on ART.

Patients who did not fulfill these criteria were classified as people without HIV.

### Outcome

The primary outcome was all-cause in-hospital mortality. Patients were followed until hospital discharge. As a secondary outcome, we also captured readmission to a medical or ICU service at a GEMINI hospital site for any reason within 30 days of discharge.

### Covariates

The following covariates were considered potential prognostic factors and collected for the study:

1. Demographics: age, sex
2. Placement: admitted from long-term care home
3. Time and setting: hospital site, admission year, admission meteorological season
4. Comorbidities: individual components of the updated Charlson comorbidity index [20], cerebrovascular disease
5. Illness severity: ICU admission within 24 hours of admission, modified Laboratory-based Acute Physiology Score within 24 hours of admission [21].

In addition, we collected CD4 T-lymphocyte count and percentage bloodwork results if they were done anytime during hospital stay. We did not have data on HIV viral load or culture results. We captured pathogens that were identified as cause for CAP based on the ICD-10-CA codes. Outpatient bloodwork such as CD4 T-lymphocyte count, CD4 T-lymphocyte percentage and HIV viral load were not available.

### Statistical Analysis

Descriptive statistics included mean (standard deviation) for normally distributed continuous variables, median (interquartile range [IQR]) for nonnormally distributed continuous variables, and count (%) for categorical variables. Balance of baseline characteristics between PWH and people without HIV were described using the absolute standardized difference of the mean with a threshold of >0.1 to denote significant imbalance [22].

For the primary outcome of in-hospital mortality, we used a competing risk model to describe time to dying in hospital or being discharged alive. Based on a cumulative incidence function, a subdistribution hazard ratio (sHR) was estimated in the Fine and Gray model [23]. A competing risk model was used because it accounted for differences in follow-up length and considered hospital discharge as a competing event rather than a censoring event that would violate the assumption of non-informative censoring [24, 25]. The sHR for time to discharge alive would be related to adjusted length of stay for patients who were discharged.

Overlap weighting of propensity scores was used to balance covariates between PWH and people without HIV, which estimated the average effect of the exposure for the population with the most overlap in covariates [26]. Propensity scores were estimated using a logistic regression of all the aforementioned covariates except for illness severity within 24 hours including ICU admission and modified Laboratory-based Acute Physiology Score because the initial illness severity covariates would be intermediate factors within the causal pathway of how HIV status affects mortality. Overlap weighting of propensity score between 2 groups leads to an exact balance in the means of included covariates, resulting in an absolute standardized difference of the mean of 0 [26, 27]. Complete case analysis was done for overlap weighting of propensity scores. The overlap weights were then included in the competing risk model to estimate the adjusted sHR.

In a preplanned subgroup analysis, we compared the outcomes of PWH with AIDS to PWH without AIDS using the same methods as described previously. This was an exploratory analysis because we anticipated smaller sample sizes especially for the HIV with AIDS group.

Reported confidence intervals (CI) were 2-sided 95% intervals. Statistical tests were 2-sided with a *P* < .05 significance level. All analyses were done with statistical software R, version 4.1.3 (R Foundation for Statistical Computing). The package PSweight was used for propensity score weighting [28].

### Data Reporting Policy

In compliance with GEMINI data policy, all cells containing or revealing 5 individuals or fewer were suppressed to protect patient confidentiality and hospital names were anonymized.

## RESULTS

From 1 January 2015 to 1 July 2022, there were 82 822 patients admitted to a GEMINI hospital site with CAP. There were 1518 (1.8%) PWH and 81 304 (98.2%) people without HIV. Exposure, follow-up and outcome data were complete for all patients.

Baseline characteristics for people with and without HIV are described in Table 1 and Supplementary Table 5. In general, PWH were more likely to be younger and have fewer comorbidities than people without HIV.

**Table 1.** Baseline Characteristics

	People With HIV (N = 1518)	People Without HIV (N = 81 304)	ASDM
Demographics			
Age mean SD	54.6 (17.0)	73.6 (16.2)	1.1450
Sex			
Female	486 (32.0%)	39 415 (48.5%)	0.3405
Male	1032 (68.0%)	41 889 (51.5%)	0.3405
From long-term care home	18 (1.2%)	4334 (5.3%)	0.2351
Admission year			
2015	140 (9.2%)	7695 (9.5%)	0.0083
2016	176 (11.6%)	11 677 (14.4%)	0.0824
2017	207 (13.6%)	12 864 (15.8%)	0.0617
2018	277 (18.3%)	14 703 (18.1%)	0.0042
2019	312 (20.6%)	14 782 (18.2%)	0.0601
2020	205 (13.5%)	10 148 (12.5%)	0.0304
2021	142 (9.4%)	6494 (8.0%)	0.0486
2022	59 (3.9%)	2941 (3.6%)	0.0142
Admission season			
Spring	391 (25.8%)	21 904 (26.9%)	0.0269
Summer	348 (22.9%)	15 367 (18.9%)	0.0991
Autumn	341 (22.5%)	17 410 (21.4%)	0.0254
Winter	438 (28.9%)	26 623 (32.8%)	0.0844
Comorbidities			
Congestive heart failure	81 (5.3%)	10 197 (12.5%)	0.2546
Chronic pulmonary disease	102 (6.7%)	3230 (4.0%)	0.1223
Connective tissue disease	≤5 (≤0.3%)	286 (0.4%)	0.0627
Mild liver disease	26 (1.7%)	405 (0.5%)	0.1164
Moderate to severe liver disease	≤5 (≤0.3%)	185 (0.2%)	0.0193
Chronic kidney disease	16 (1.1%)	1273 (1.6%)	0.0450
Complicated diabetes mellitus	27 (1.8%)	2925 (3.6%)	0.1126
Malignancy	39 (2.6%)	1802 (2.2%)	0.0231
Metastatic cancer	10 (0.7%)	807 (1.0%)	0.0369
Dementia	6 (0.4%)	2683 (3.3%)	0.2170
Hemiplegia	≤5 (≤0.3%)	116 (0.1%)	0.0535
Cerebrovascular disease	≤5 (≤0.3%)	195 (0.2%)	0.0251
Illness severity			
ICU admission within 24 h	163 (10.7%)	6704 (8.3%)	0.0851
mLAPS score within 24 h mean (SD)	29.8 (23.6) N = 1432	29.5 (20.0) N = 73 762	0.0105

Hospital sites data are reported in Supplementary Table 5.

Abbreviations: ASDM, absolute standardized difference of the mean; HIV, human immunodeficiency virus; ICU, intensive care unit; mLAPS, modified Laboratory-based Acute Physiology Score; SD, standard deviation.

### Microbiology and Empiric Antibiotic Therapy

The ICD-10 diagnosis codes identified an organism in 224 (14.8%) PWH patients and 12 776 (15.7%) people without HIV (Supplementary Table 6). The most common viral pathogen was influenza that was found in 117 (7.7%) PWH and 7447 (9.2%) people without HIV. *S. pneumoniae* was found in 23 (1.5%) PWH and 499 (0.6%) people without HIV.

Complete data for empiric antibiotic therapy defined as antibiotics initiated within 48 hours of admission is described in Supplementary Table 7. The proportion of patients on antibiotic coverage for methicillin-resistant *S. aureus* was 9.8% in PWH and 4.1% in people without HIV. The proportion of patients on antibiotic coverage against *Pseudomonas aeruginosa* was 19.0% in PWH and 13.4% in people without HIV.

### Outcomes

**Figure 1.**
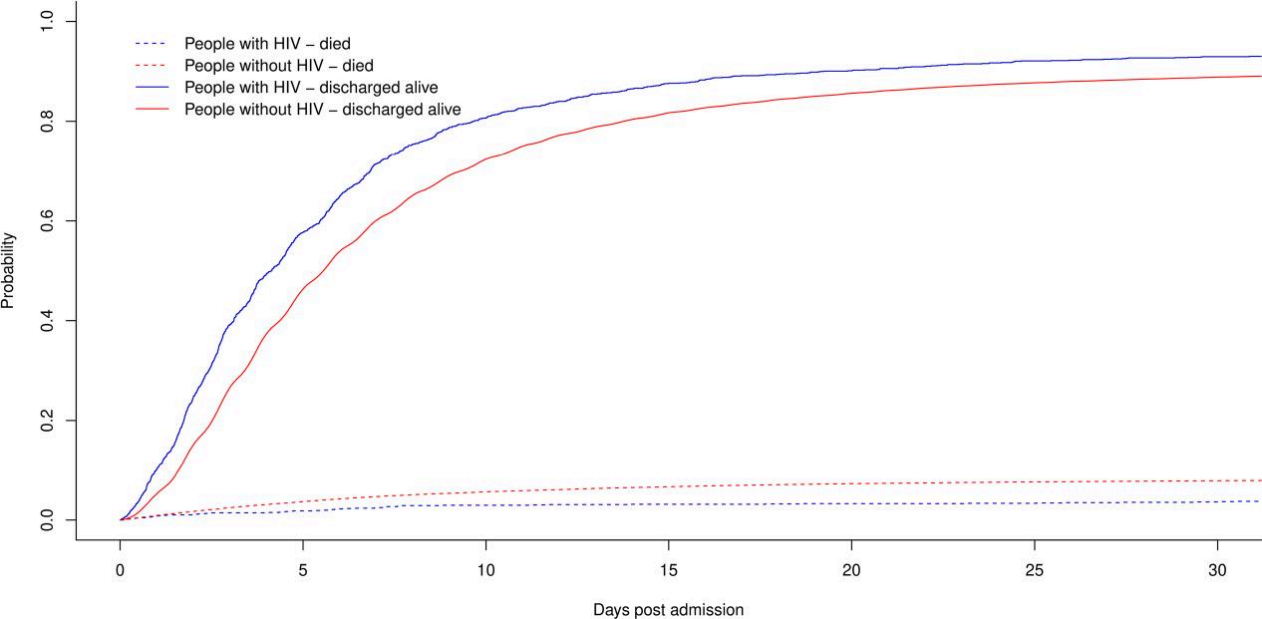
Cumulative incidence function of time to death in hospital or discharge alive in people with HIV versus people without HIV. HIV, human immunodeficiency virus.

The unadjusted median (IQR) length of stay was 3.9 (2.0–7.2) days in PWH and 5.0 (2.7–9.2) days in people without HIV. In hospital, 67 (4.4%) PWH and 6873 (8.5%) people without HIV died (Figure 1). In a competing risk model, PWH had an sHR of 0.51 (95% CI, .40–.65; *P* < .0001) for dying in hospital and 1.33 (95% CI, 1.27–1.41; *P* < .0001) for being discharged alive when compared to people without HIV.

Of those who survived to discharge, 136 (9.4%) of 1451 PWH and 10 351 (13.9%) of 74 431 people without HIV were readmitted to a GEMINI hospital site within 30 days (*P* < .0001). In particular, ≤5 (≤0.3%) of 1451 PWH and 251 (0.3%) of 74 431 people without HIV were readmitted with diagnosis of pneumonia and subsequently died in hospital (*P* = .2506).

### Overlap Weighting of Propensity Score

After propensity score weighting, the overlap population is described in Table 2 and Supplementary Table 8. The absolute standardized difference of the mean was 0 for all covariates listed in Table 2 after overlap weighting. PWH had an adjusted sHR of 1.02 (95% CI, .80–1.31 *P* = .8440) for dying in hospital and 1.00 (95% CI, .95–1.06 *P* = .8740) for being discharged alive when compared to people without HIV.

**Table 2.** Balance of Baseline Characteristics After Overlap Weighting Using Propensity Scores

	People With HIV Effective Sample Size of 1508.4	People Without HIV Effective Sample Size of 21 180.3
Demographics		
Age mean SD	55.5 (16.8)	55.5 (20.1)
Sex		
Female	32.8%	32.8%
Male	67.2%	67.2%
From long-term care home	1.2%	1.2%
Admission year		
2015	9.1%	9.1%
2016	11.8%	11.8%
2017	13.6%	13.6%
2018	18.2%	18.2%
2019	20.7%	20.7%
2020	13.5%	13.5%
2021	9.1%	9.1%
2022	3.9%	3.9%
Admission season		
Spring	25.8%	25.8%
Summer	22.7%	22.7%
Autumn	22.4%	22.4%
Winter	29.1%	29.1%
Comorbidities		
Congestive heart failure	5.6%	5.6%
Chronic pulmonary disease	6.7%	6.7%
Connective tissue disease	0.1%	0.1%
Mild liver disease	1.6%	1.6%
Moderate to severe liver disease	0.3%	0.3%
Chronic kidney disease	1.1%	1.1%
Complicated diabetes mellitus	1.9%	1.9%
Malignancy	2.5%	2.5%
Metastatic cancer	0.7%	0.7%
Dementia	0.4%	0.4%
Hemiplegia	0%	0%
Cerebrovascular disease	0.1%	0.1%

Hospital sites data are reported in Supplementary Table 8.

Abbreviations: HIV, human immunodeficiency virus; SD, standard deviation.

### Subgroup Analysis of HIV With or Without AIDS

Of 1518 PWH, 440 (29.0%) patients had a diagnosis of AIDS and 1078 (71.0%) patients did not have a diagnosis of AIDS (Supplementary Table 9). Bloodwork for CD4 T-lymphocyte percentage and/or count was drawn during hospital admission for 328 (21.6%) PWH including 182 PWH with AIDS and 146 PWH without AIDS. The median (IQR) CD4 T-lymphocyte percentage was 15% (6%–25%) in PWH with AIDS and 22% (12%–29%) in PWH without AIDS. The median (IQR) absolute CD4 T-lymphocyte count was 120 (29–293) cells/mm^3^ in PWH with AIDS and 211 (100–419) cells/mm^3^ in PWH without AIDS.

**Figure 2.**
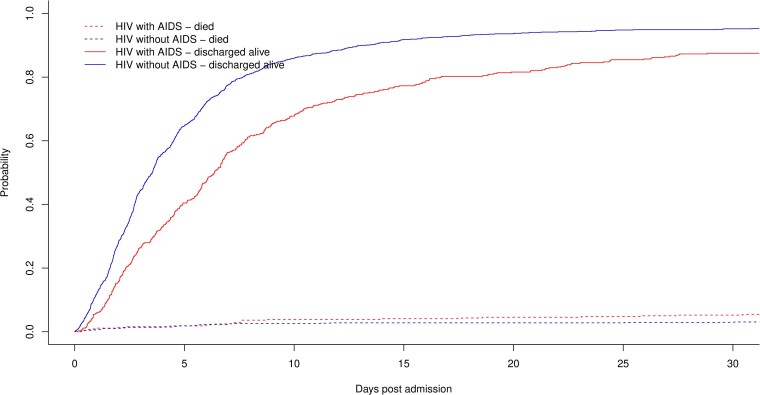
Cumulative incidence function of time to death in hospital or discharge alive in people with HIV who had AIDS versus people with HIV who did not have AIDS. HIV, human immunodeficiency virus.

The unadjusted median (IQR) length of stay was 6.1 (2.7–11.0) days in PWH with AIDS and 3.4 (1.8–6.1) days in PWH without AIDS. In the hospital, 32 (7.3%) PWH with AIDS and 35 (3.3%) PWH without AIDS died, respectively (Figure 2). In a competing risk model, PWH with AIDS had an sHR of 2.33 (95% CI, 1.44–3.77; *P* = .0006) for dying in the hospital and 0.60 (95% CI, .54–.68; *P* < .0001) for being discharged alive when compared to PWH without AIDS. The population after overlap weighting using propensity scores is described in Supplementary Table 10. In this overlap population, PWH with AIDS had an adjusted sHR of 3.04 (95% CI, 1.69–5.45; *P* = .0002) for dying in the hospital and 0.57 (95% CI, .50–.65; *P* < .0001) for being discharged alive when compared to PWH without AIDS.

Of those who survived to discharge, 71 (17.4%) of 408 PWH and 65 (6.2%) of 1043 PWH without AIDS were readmitted to a GEMINI hospital site within 30 days (*P* < .0001). In particular, ≤5 of 408 (≤1.2%) PWH with AIDS and ≤5 of 1043 (≤0.5%) PWH without AIDS were admitted to a GEMINI hospital site for pneumonia and subsequently died in the hospital (*P* = .4834).

## DISCUSSION

In this large multicenter retrospective study of 1518 PWH and 81 304 people without HIV admitted for CAP, PWH had a numerically lower in-hospital mortality with an adjusted sHR of 1.02 (95% CI, .80–1.31; *P* = .8440). The sHR point estimate and CI suggest that the mortality risk for PWH is not significantly different from people without HIV. Within the PWH group, patients without AIDS had a significantly lower mortality risk than patients with AIDS.

Our study finding of similar mortality risk for PWH and people without HIV admitted for CAP is consistent with prior studies. In a case-control study of patients with pneumococcal pneumonia, 50 PWH on ART with CD4 T-lymphocyte count of >350 cells/mm^3^ were matched to 100 control patients by age, sex, and comorbidities [10]. The 30-day mortality was 0% in both groups [10]. The largest study to date used a Veterans Aging Cohort database of only males aged ≥50 years to compare between 670 PWH and 533 people without HIV admitted for CAP [11]. The 30-day mortality was 5.5% in PWH and 5.1% in people without HIV [11]. In an adjusted model, there was no difference between the 2 groups in terms of 30-day mortality, length of stay, or readmission [11]. In contrast to these 2 studies, our study included all adult patients admitted with CAP regardless of microbiologic etiology, age, and sex. Furthermore, our study had more than twice the sample size of the largest study to date [11]. Last, our study was conducted in 31 academic and community hospitals that captured approximately 50% of inpatient beds in Ontario, making the results more likely to be widely generalizable.

Our study had several limitations. First, outpatient CD4 T-lymphocyte count, HIV viral load, and outpatient ART before admission were not available, so we could not ascertain which patients were virally suppressed on ART. Inpatient CD4 T-lymphocyte count likely underestimated the baseline CD4 T-lymphocyte count before admission because CAP generally leads to significantly depressed total lymphocyte and CD4 T-lymphocyte count [29]. However, the CD4/CD8 T-lymphocyte ratio tends to remain similar, suggesting that the CD4 T-lymphocyte percentage may remain constant [29]. As a subgroup analysis, we classified PWH based on physician diagnosis of AIDS, which has not been validated. A negative AIDS status likely correlated with effective ART and HIV viral suppression because ART would preserve CD4 T-lymphocyte count and prevent AIDS-defined illness in most PWH. Accordingly, the CD4 T-lymphocyte percentage was much lower in PWH with AIDS in our study. The study classification based on AIDS seemed to be clinically relevant and important, because PWH with AIDS had a significantly higher mortality risk. Any misclassification of AIDS status would make the HIV with AIDS and HIV without AIDS groups more similar, bias toward the null, and make our estimates more conservative.

Second, only a small proportion of patients had an identified pathogen based on ICD-10-CA codes, which was likely from poor sensitivity of ICD-10-CA diagnosis codes. Microbiology data were not available in our study. However, most CAP cases are treated empirically without a microbiologic diagnosis in clinical practice, and current guidelines do not recommend any microbiologic workup in patients admitted with nonsevere CAP without any risk factors [12]. Even in prospective studies with thorough and systematic microbiologic workup of CAP, a pathogen was found in only 30% of cases [30]. Because of the lack of data on microbiology and risk factors for methicillin-resistant *S. aureus* and *Pseudomonas aeruginosa*, we cannot judge the appropriateness of antibiotic coverage for these 2 organisms. Prior microbiologic studies showed that HIV status did not significantly increase the risk of CAP due to *S. aureus* or *P. aeruginosa* [31, 32]. Therefore, future studies may focus on the appropriateness of empiric antibiotic CAP treatment in PWH because this could be an opportunity for antimicrobial stewardship.

Third, follow-up ended on hospital discharge, so we reported only in-hospital mortality. In-hospital mortality should have captured most deaths attributable to CAP. Our in-hospital mortality of 4.4% is either higher than or close to the 30-day all-cause mortality reported for PWH admitted with CAP in prior studies [10, 11], suggesting that we did not miss a significant number of deaths. Furthermore, patients who were discharged, deteriorated, and then readmitted to a GEMINI hospital site would still be captured in the secondary outcome of readmission. Readmission to a GEMINI hospital site should capture the vast majority of readmissions, because more than 80% of readmissions in our region occur at the same hospital [33] and GEMINI hospital sites make up approximately half of all acute-care hospital beds in Ontario.

Fourth, as is the case for all observational studies, there may be residual confounding. Our study did not collect or capture potential confounders such as ethnicity, body mass index, smoking history, or vaccination status. Nevertheless, we had adjusted for many prognostic factors such as demographics, setting, and comorbidities using propensity score weighting.

Our study findings have 2 important implications. First, HIV status by itself should not be considered a poor prognostic factor when risk stratifying CAP regarding decisions on hospital or ICU admission or discharge. As supported from findings of a previous study [11], PWH, especially if virologically suppressed on ART with a normal CD4 T-lymphocyte count, can be regarded as the same risk group as people without HIV. Second, among PWH admitted for CAP, AIDS diagnosis significantly increased risk of death. It illustrates the importance of early and timely initiation of ART in PWH to prevent progression to AIDS, which could then potentially prevent death from common infections such as CAP. Furthermore, PWH with AIDS may particularly benefit from being up-to-date on influenza and pneumococcal vaccination.

In conclusion, our study adds to the evidence that HIV infection by itself is not a risk factor for mortality in CAP in the current era of effective ART. An explicit statement of this in new CAP guidelines may help guide clinicians when they risk stratify and manage PWH with CAP.

## Supplementary Data

Supplementary materials are available at *Clinical Infectious Diseases* online. Consisting of data provided by the authors to benefit the reader, the posted materials are not copyedited and are the sole responsibility of the authors, so questions or comments should be addressed to the corresponding author.

## Supplementary Material

ciae393_Supplementary_Data

## Notes

***Author contributions.*** A. D. B. and A. A. V. had full access to the data and take responsibility for data and analysis. A. D. B. and A. A. V. conceived and designed the study. A. D. B. performed the data analysis. All authors contributed to the interpretation of results. A. D. B. wrote the first draft of the manuscript. All authors contributed to the revision of manuscript drafts. S. S., A. A. V., and F. R. supervised the study and provided administrative, technical, and material support.

***Data availability.*** Data can be made available upon reasonable request to amol.verma@mail.utoronto.ca.

***Financial support*.** No funding was received for this study.
