# Supplementary material for: Community-acquired Pneumonia in People With HIV During the Current Era of Effective Antiretroviral Therapy: A Multicenter Retrospective Cohort Study
Source: Clin Infect Dis. 2024 Jul 27;80(2):397–403. doi: 10.1093/cid/ciae393 (PMC11848276; doi:10.1093/cid/ciae393)
Supplement: ciae393_Supplementary_Data [file ciae393_supplementary_data.docx]

Supplement Table 1. Description of hospital sites

| Academic or community | Focus | Bed-size | Infectious diseases service available |
| --- | --- | --- | --- |
| Community | General | 500-750 beds | Yes |
| Community | General | 250-500 beds | Yes |
| Academic | Trauma center  Neurosurgery and stroke  Cardiac surgery | 1,000-1,250 beds | Yes |
| Academic | Cancer center | 250-500 beds | Yes |
| Community | General | 500-750 beds | Yes |
| Academic | General | 250-500 beds | Yes |
| Academic | General | 250-500 beds | Yes |
| Academic | General | 500-750 beds | Yes |
| Community | Chronic kidney disease program | 500-750 beds | Yes |
| Community | Stroke centre | 250-500 beds | Yes |
| Academic | Centre of excellence for obstetrics medicine, gastroenterology, surgical oncology | 250-500 beds | Yes |
| Community | General | 1,000-1,250 beds combined | Yes |
| Community | General |  | Yes |
| Community | General |  | Yes |
| Community | Affiliated teaching hospital  Obstetrical centre | 250-500 beds | Yes |
| Community | General | 250-500 beds | Yes |
| Community | General | 250-500 beds | Yes |
| Community | General | <250 beds | Yes |
| Academic | Trauma center  Veterans care facility | 1,250-1,500 beds | Yes |
| Community | General | 250-500 beds | Yes |
| Community | Affiliated teaching hospital  General | 500-750 beds | Yes |
| Community | General | 250-500 beds | Yes |
| Community | General | 750-1,000 beds | Yes |
| Academic | Trauma centre  Centre of excellence for care of homeless and disadvantaged | 250-500 beds | Yes |
| Academic | General | 250-500 beds | Yes |
| Academic | Centre of excellence for cardiology, cardiac surgery and solid organ transplant | 250-500 beds | Yes |
| Academic | Centre of excellence for neurosurgery and neurology | 250-500 beds | Yes |
| Academic | Cancer centre | <250 beds | Yes |
| Community | General | 500-750 beds | Yes |
| Community | General | 250-500 beds | Yes |
| Community | Rehabilitation | <250 beds | Yes |

Table 2. STROBE checklist for cohort studies

Note that page numbers referred to manuscript pages prior to journal publication formatting

|  | Item No | Recommendation | Page No |
| --- | --- | --- | --- |
| **Title and abstract** | 1 | (*a*) Indicate the study’s design with a commonly used term in the title or the abstract | 1 |
|  |  | (*b*) Provide in the abstract an informative and balanced summary of what was done and what was found | 2,3 |
| Introduction | | | |
| Background/rationale | 2 | Explain the scientific background and rationale for the investigation being reported | 4 |
| Objectives | 3 | State specific objectives, including any prespecified hypotheses | 4 |
| Methods | | | |
| Study design | 4 | Present key elements of study design early in the paper | 5 |
| Setting | 5 | Describe the setting, locations, and relevant dates, including periods of recruitment, exposure, follow-up, and data collection | 5 |
| Participants | 6 | (*a*) Give the eligibility criteria, and the sources and methods of selection of participants. Describe methods of follow-up | 5 |
|  |  | (*b*) For matched studies, give matching criteria and number of exposed and unexposed |  |
| Variables | 7 | Clearly define all outcomes, exposures, predictors, potential confounders, and effect modifiers. Give diagnostic criteria, if applicable | 6,7 |
| Data sources/ measurement | 8* | For each variable of interest, give sources of data and details of methods of assessment (measurement). Describe comparability of assessment methods if there is more than one group | 6,7 |
| Bias | 9 | Describe any efforts to address potential sources of bias | 7,8 |
| Study size | 10 | Explain how the study size was arrived at | 5 |
| Quantitative variables | 11 | Explain how quantitative variables were handled in the analyses. If applicable, describe which groupings were chosen and why | 7,8 |
| Statistical methods | 12 | (*a*) Describe all statistical methods, including those used to control for confounding | 7 |
|  |  | (*b*) Describe any methods used to examine subgroups and interactions | 8,9 |
|  |  | (*c*) Explain how missing data were addressed | 8 |
|  |  | (*d*) If applicable, explain how loss to follow-up was addressed | 8 |
|  |  | (*e*) Describe any sensitivity analyses | N/A |
| Results | | |  |
| Participants | 13* | (a) Report numbers of individuals at each stage of study—eg numbers potentially eligible, examined for eligibility, confirmed eligible, included in the study, completing follow-up, and analysed | 10 |
|  |  | (b) Give reasons for non-participation at each stage | 10 |
|  |  | (c) Consider use of a flow diagram | N/A |
| Descriptive data | 14* | (a) Give characteristics of study participants (eg demographic, clinical, social) and information on exposures and potential confounders | Tab1 |
|  |  | (b) Indicate number of participants with missing data for each variable of interest | Tab1 |
|  |  | (c) Summarise follow-up time (eg, average and total amount) | 10 |
| Outcome data | 15* | Report numbers of outcome events or summary measures over time | 10 |

| Main results | 16 | (*a*) Give unadjusted estimates and, if applicable, confounder-adjusted estimates and their precision (eg, 95% confidence interval). Make clear which confounders were adjusted for and why they were included | 11 |
| --- | --- | --- | --- |
|  |  | (*b*) Report category boundaries when continuous variables were categorized | N/A |
|  |  | (*c*) If relevant, consider translating estimates of relative risk into absolute risk for a meaningful time period | N/A |
| Other analyses | 17 | Report other analyses done—eg analyses of subgroups and interactions, and sensitivity analyses | 11,12 |
| Discussion | | | |
| Key results | 18 | Summarise key results with reference to study objectives | 13 |
| Limitations | 19 | Discuss limitations of the study, taking into account sources of potential bias or imprecision. Discuss both direction and magnitude of any potential bias | 13-15 |
| Interpretation | 20 | Give a cautious overall interpretation of results considering objectives, limitations, multiplicity of analyses, results from similar studies, and other relevant evidence | 13,15 |
| Generalisability | 21 | Discuss the generalisability (external validity) of the study results | 13 |
| Other information | | | |
| Funding | 22 | Give the source of funding and the role of the funders for the present study and, if applicable, for the original study on which the present article is based | 17 |

*Give information separately for exposed and unexposed groups.

**Note:** An Explanation and Elaboration article discusses each checklist item and gives methodological background and published examples of transparent reporting. The STROBE checklist is best used in conjunction with this article (freely available on the Web sites of PLoS Medicine at http://www.plosmedicine.org/, Annals of Internal Medicine at http://www.annals.org/, and Epidemiology at http://www.epidem.com/). Information on the STROBE Initiative is available at http://www.strobe-statement.org.

Supplement Table 3. ICD-10-CA codes for HIV and AIDS

| Classification | ICD-10-CA codes | Definition |
| --- | --- | --- |
| HIV with AIDS | B20.0 | HIV resulting in mycobacterial infection including tuberculosis |
|  | B20.2 | HIV disease resulting in cytomegaloviral disease |
|  | B20.4 | HIV disease resulting in candidiasis |
|  | B20.6 | HIV disease resulting in *Pneumocystis jirovecii* pneumonia |
|  | B21.0 | HIV disease resulting in Kaposi sarcoma |
|  | B21.1 | HIV disease resulting in Burkitt lymphoma |
|  | B21.2 | HIV disease resulting in other types of non-Hodgkin lymphoma |
|  | B22.0 | HIV disease resulting in encephalopathy |
|  | B22.2 | HIV disease resulting in wasting syndrome |
|  | B24 | Unspecified HIV disease including AIDS not otherwise specified or AIDS-related complex not otherwise specified |
|  | F02.4 | Dementia in HIV disease |
| HIV without AIDS | B20.1 | HIV disease resulting in other bacterial infections |
|  | B20.3 | HIV disease resulting in other viral infections |
|  | B20.5 | HIV disease resulting in other mycoses |
|  | B20.7 | HIV disease resulting in multiple infections |
|  | B20.8 | HIV disease resulting in other infectious and parasitic diseases |
|  | B20.9 | HIV disease resulting in unspecified infectious or parasitic disease |
|  | B21.3 | HIV disease resulting in other malignant neoplasm of lymphoid, haematopoietic and related tissue |
|  | B21.7 | HIV disease resulting in multiple malignant neoplasms |
|  | B21.8 | HIV disease resulting in other malignant neoplasms |
|  | B21.9 | HIV disease resulting in unspecified malignant neoplasm |
|  | B22.1 | HIV disease resulting in lymphoid interstitial pneumonitis |
|  | B22.7 | HIV disease resulting in multiple diseases classified elsewhere |
|  | B23.x | HIV disease resulting in other conditions |
|  | Z21 | Asymptomatic HIV infection status |
|  | O98.7 | HIV disease complicating pregnancy, childbirth and the puerperium |

Supplement Table 4. HIV specific anti-retroviral medications

| Class | Medications |
| --- | --- |
| Nucleoside Reverse Transcriptase Inhibitor (NRTI) | Abacavir, Zidovudine |
| Non-Nucleoside Reverse Transcriptase Inhibitor (NNRTI) | Doravirine, Efavirenz, Etravirine, Nevirapine, Rilpivirine |
| Integrase inhibitors | Cabotegravir, Dolutegravir, Raltegravir, Elvitegravir, Bictegravir |
| Protease inhibitors | Atazanavir, Darunavir, Fosamprenavir, Lopinavir, Ritonavir, Tipranavir |
| Fusion inhibitors | Enfuviritide |
| Entry inhibitors | Maraviroc |
| Other | Fostemsavir, Ibalizumab, Lenacapavir, Cobicistat |
| Combinations Brand Names | Biktarvy, Dovato, Juluca, Delstrigo, Genvoya, Stribild, Complera  Atripla, Cimduo, Combivir, Kaletra, Triumeq, Kivexa, |

Lamivudine, Tenofovir, or Tenofovir/Emtricitabine (Truvada or Descovy) alone by itself is not included in this list, because there may be alternative indications such as Hepatitis B treatment or HIV pre-exposure prophylaxis. People with HIV taking Lamivudine, Tenofovir, or Tenofovir/Emtricitabine should be on another anti-retroviral medication from the list above for treatment.

There were no patients taking HIV medications for the indication of post-exposure prophylaxis in this study.

Supplement Table 5. Hospital sites

|  | People with HIV  (N=1,518) | People without HIV  (N=81,304) | ASDM |
| --- | --- | --- | --- |
| Hospital site |  |  |  |
| 1 | 137 (9.0%) | 2,678 (3.3%) | 0.2401 |
| 2 | 55 (3.6%) | 2,368 (2.9%) | 0.0400 |
| 3 | ≤5 (≤0.3%) | 104 (0.1%) | 0.0199 |
| 4 | 217 (14.3%) | 2,264 (2.8%) | 0.4209 |
| 5 | 77 (5.1%) | 3,521 (4.3%) | 0.0351 |
| 6 | 41 (2.7%) | 2,811 (3.5%) | 0.0438 |
| 7 | 24 (1.6%) | 2,969 (3.7%) | 0.1300 |
| 8 | 41 (2.7%) | 2,857 (3.5%) | 0.0469 |
| 9 | 67 (4.4%) | 1,678 (2.1%) | 0.1330 |
| 10 | 44 (2.9%) | 2,904 (3.6%) | 0.0381 |
| 11 | 24 (1.6%) | 4,154 (5.1%) | 0.1972 |
| 12 | 20 (1.3%) | 1,814 (2.2%) | 0.0692 |
| 13 | 48 (3.2%) | 3,926 (4.8%) | 0.0852 |
| 14 | 8 (0.5%) | 1,640 (2.0%) | 0.1333 |
| 15 | 41 (2.7%) | 3,518 (4.3%) | 0.0884 |
| 16 | ≤5 (≤0.3%) | 294 (0.4%) | 0.0463 |
| 17 | 32 (2.1%) | 2,022 (2.5%) | 0.0253 |
| 18 | 26 (1.7%) | 1,413 (1.7%) | 0.0019 |
| 19 | 28 (1.8%) | 1,828 (2.3%) | 0.0285 |
| 20 | 90 (5.9%) | 4,399 (5.4%) | 0.0224 |
| 21 | 63 (4.2%) | 3,331 (4.1%) | 0.0027 |
| 22 | 60 (4.0%) | 4,534 (5.6%) | 0.0763 |
| 23 | 15 (1.0%) | 2,117 (2.6%) | 0.1219 |
| 24 | 35 (2.3%) | 2,831 (3.5%) | 0.0702 |
| 25 | 94 (6.2%) | 4,089 (5.0%) | 0.0506 |
| 26 | 66 (4.4%) | 2,387 (2.9%) | 0.0754 |
| 27 | 24 (1.6%) | 1,195 (1.5%) | 0.0091 |
| 28 | 24 (1.6%) | 2,651 (3.3%) | 0.1094 |
| 29 | 80 (5.3%) | 5,196 (6.4%) | 0.0478 |
| 30 | 34 (2.2%) | 3,326 (4.1%) | 0.1059 |
| 31 | ≤5 (≤0.3%) | 485 (0.6%) | 0.1096 |

ASDM = absolute standardized difference of the mean

Supplement Table 6. Pathogens based on ICD-10-CA codes

|  | People with HIV  (N=1,518) | People without HIV  (N=81,304) |
| --- | --- | --- |
| Virus |  |  |
| Influenza | 117 (7.7%) | 7,447 (9.2%) |
| Respiratory syncytial virus | 9 (0.6%) | 1,158 (1.4%) |
| Other respiratory viruses | 29 (1.9%) | 1,740 (2.1%) |
| Typical bacterial organisms |  |  |
| *Streptococcus pneumoniae* | 23 (1.5%) | 499 (0.6%) |
| Other Streptococcus species | 11 (0.7%) | 259 (0.3%) |
| *Haemophilus influenza* | 6 (0.4%) | 168 (0.2%) |
| Atypical bacterial organisms |  |  |
| *Chlamydia pneumoniae* | ≤5 (≤0.3%) | ≤5 (≤0.01%) |
| *Mycoplasma pneumoniae* | ≤5 (≤0.3%) | 42 (0.1%) |
| *Legionella* | ≤5 (≤0.3%) | ≤5 (≤0.01%) |
| Other bacterial organisms |  |  |
| *Staphylococcus* species | 18 (1.2%) | 578 (0.7%) |
| *Escherichia coli* | ≤5 (≤0.3%) | 90 (0.1%) |
| *Klebsiella* species | ≤5 (≤0.3%) | 185 (0.2%) |
| *Pseudomonas aeruginosa* | ≤5 (≤0.3%) | 518 (0.6%) |
| Other Gram negative | ≤5 (≤0.3%) | 90 (0.1%) |

Supplement Table 7. Empiric antibiotic therapy for CAP

|  | People with HIV  (N=1,234) | People without HIV  (N=62,060) |
| --- | --- | --- |
| First line or alternative anti-bacterial agents |  |  |
| Beta-lactam  (Ceftriaxone, Cefotaxime or Amoxicillin-Clavulanate) | 827 (67.0%) | 35,414 (57.1%) |
| Macrolide | 683 (55.4%) | 25,586 (41.2%) |
| Doxycycline | 24 (1.9%) | 1,894 (3.1%) |
| Respiratory fluoroquinolone | 185 (15.0%) | 13,716 (22.1%) |
| Additional coverage |  |  |
| Anti-MRSA coverage based on ATS/IDSA 2019  guidelines  (Vancomycin IV or Linezolid) | 121 (9.8%) | 2,555 (4.1%) |
| Anti-Pseudomonal coverage based on ATS/IDSA 2019  guidelines  (Piperacillin-Tazobactam, Cefepime, Ceftazidime,  Imipenem, Meropenem or Aztreonam) | 235 (19.0%) | 8,317 (13.4%) |

Complete medication record was not available in 7 hospital sites, so antibiotic data is only available for 63,294 patients.

Supplement Table 8. Hospital sites after overlap weighting using propensity scores

|  | People with HIV  Effective sample size of 1,508.4 | People without HIV  Effective sample size of 21,180.3 |
| --- | --- | --- |
| Hospital site |  |  |
| 1 | 8.7% | 8.7% |
| 2 | 3.6% | 3.6% |
| 3 | 0.1% | 0.1% |
| 4 | 12.7% | 12.7% |
| 5 | 5.1% | 5.1% |
| 6 | 2.8% | 2.8% |
| 7 | 1.7% | 1.7% |
| 8 | 2.8% | 2.8% |
| 9 | 4.2% | 4.2% |
| 10 | 3.0% | 3.0% |
| 11 | 1.7% | 1.7% |
| 12 | 1.4% | 1.4% |
| 13 | 3.3% | 3.3% |
| 14 | 0.6% | 0.6% |
| 15 | 2.8% | 2.8% |
| 16 | 0.1% | 0.1% |
| 17 | 2.2% | 2.2% |
| 18 | 1.7% | 1.7% |
| 19 | 1.9% | 1.9% |
| 20 | 6.1% | 6.1% |
| 21 | 4.2% | 4.2% |
| 22 | 4.1% | 4.1% |
| 23 | 1.0% | 1.0% |
| 24 | 2.4% | 2.4% |
| 25 | 6.3% | 6.3% |
| 26 | 4.3% | 4.3% |
| 27 | 1.6% | 1.6% |
| 28 | 1.7% | 1.7% |
| 29 | 5.4% | 5.4% |
| 30 | 2.4% | 2.4% |
| 31 | 0% | 0% |

Supplement Table 9. Baseline characteristics for people with HIV who had AIDS and people with HIV who did not have AIDS

|  | People with HIV and AIDS  (N=440) | People with HIV and without AIDS  (N=1,078) | ASDM |
| --- | --- | --- | --- |
| Demographics |  |  |  |
| Age mean SD | 49.3 (12.5) | 56.8 (18.0) | 0.4869 |
| Sex |  |  |  |
| Female | 100 (22.7%) | 386 (35.8%) | 0.2905 |
| Male | 340 (77.3%) | 692 (64.2%) | 0.2905 |
| From long-term care home | 9 (2.1%) | 9 (0.8%) | 0.1017 |
| Hospital site |  |  |  |
| 1 | 70 (15.9%) | 67 (6.2%) | 0.3128 |
| 2 | 26 (5.9%) | 29 (2.7%) | 0.1592 |
| 3 | ≤5 (≤1.1%) | ≤5 (≤0.5%) | 0.0675 |
| 4 | 61 (13.9%) | 156 (14.5%) | 0.0174 |
| 5 | 18 (4.1%) | 59 (5.5%) | 0.0648 |
| 6 | 7 (1.6%) | 34 (3.2%) | 0.1028 |
| 7 | ≤5 (≤1.1%) | 22 (2.0%) | 0.1433 |
| 8 | 9 (2.1%) | 32 (3.0%) | 0.0591 |
| 9 | 17 (3.9%) | 50 (4.6%) | 0.0384 |
| 10 | 10 (2.3%) | 34 (3.2%) | 0.0543 |
| 11 | 6 (1.4%) | 18 (1.7%) | 0.0250 |
| 12 | 6 (1.4%) | 14 (1.3%) | 0.0057 |
| 13 | 20 (4.6%) | 28 (2.6%) | 0.1051 |
| 14 | 6 (1.4%) | ≤5 (≤0.5%) | 0.1347 |
| 15 | 6 (1.4%) | 35 (3.3%) | 0.1257 |
| 16 | ≤5 (≤1.1%) | ≤5 (≤0.5%) | 0.0610 |
| 17 | 7 (1.6%) | 25 (2.3%) | 0.0526 |
| 18 | 14 (3.2%) | 12 (1.1%) | 0.1431 |
| 19 | 17 (3.9%) | 11 (1.0%) | 0.1850 |
| 20 | 16 (3.6%) | 74 (6.9%) | 0.1451 |
| 21 | ≤5 (≤1.1%) | 60 (5.6%) | 0.2836 |
| 22 | 7 (1.6%) | 53 (4.9%) | 0.1883 |
| 23 | 4 (0.9%) | 11 (1.0%) | 0.0114 |
| 24 | ≤5 (≤1.1%) | 32 (3.0%) | 0.1715 |
| 25 | 39 (8.9%) | 55 (5.1%) | 0.1480 |
| 26 | 19 (4.3%) | 47 (4.4%) | 0.0020 |
| 27 | 8 (1.8%) | 16 (1.5%) | 0.0262 |
| 28 | ≤5 (≤1.1%) | 22 (2.0%) | 0.1433 |
| 29 | 27 (6.1%) | 53 (4.9%) | 0.0534 |
| 30 | 9 (2.1%) | 25 (2.3%) | 0.0187 |
| 31 | ≤5 (≤1.1%) | ≤5 (≤0.5%) | 0 |
| Admission year |  |  |  |
| 2015 | 55 (12.5%) | 85 (7.9%) | 0.1530 |
| 2016 | 45 (10.2%) | 131 (12.2%) | 0.0611 |
| 2017 | 60 (13.6%) | 147 (13.6%) | 0 |
| 2018 | 73 (16.6%) | 204 (18.9%) | 0.0611 |
| 2019 | 96 (21.8%) | 216 (20.0%) | 0.0438 |
| 2020 | 51 (11.6%) | 154 (14.3%) | 0.0804 |
| 2021 | 48 (10.9%) | 94 (8.7%) | 0.0736 |
| 2022 | 12 (2.7%) | 47 (4.4%) | 0.0884 |
| Admission season |  |  |  |
| Spring | 107 (24.3%) | 284 (26.4%) | 0.0466 |
| Summer | 104 (23.6%) | 244 (22.6%) | 0.0238 |
| Autumn | 113 (25.7%) | 228 (21.2%) | 0.1072 |
| Winter | 116 (26.4%) | 322 (30.0%) | 0.0781 |
| Comorbidities |  |  |  |
| Congestive heart failure | ≤5 (≤1.1%) | 76 (7.1%) | 0.3019 |
| Chronic pulmonary disease | 57 (13.0%) | 45 (4.2%) | 0.3177 |
| Connective tissue disease | ≤5 (≤1.1%) | ≤5 (≤0.5%) | 0.0431 |
| Mild liver disease | 17 (3.9%) | 9 (0.8%) | 0.2010 |
| Moderate to severe liver disease | ≤5 (≤1.1%) | ≤5 (≤0.5%) | 0.0292 |
| Chronic kidney disease | 6 (1.4%) | 10 (0.9%) | 0.0410 |
| Complicated diabetes mellitus | ≤5 (≤1.1%) | 22 (2.0%) | 0.0724 |
| Malignancy | 27 (6.1%) | 12 (1.1%) | 0.2712 |
| Metastatic cancer | ≤5 (≤1.1%) | 7 (0.7%) | 0.0040 |
| Dementia | ≤5 (≤1.1%) | ≤5 (≤0.5%) | 0.0403 |
| Hemiplegia | ≤5 (≤1.1%) | ≤5 (≤0.5%) | 0 |
| Cerebrovascular disease | ≤5 (≤1.1%) | ≤5 (≤0.5%) | 0.0337 |
| Illness severity |  |  |  |
| ICU admission within 24 hours | 50 (11.4%) | 113 (10.5%) | 0.0283 |
| mLAPS score within 24 hours mean (SD) | 32.6 (25.1)  N=425 | 28.6 (22.9)  N=1,007 | 0.1644 |

ASDM = absolute standardized difference of the mean; ICU = intensive care unit; mLAPS = modified Laboratory-based Acute Physiology Score; SD = standard deviation

Supplement Table 10. Balance of baseline characteristics after overlap weighting using propensity scores

|  | People with HIV and AIDS  Effective sample size of 383.8 | People with HIV and without AIDS  Effective sample size of 691.8 |
| --- | --- | --- |
| Demographics |  |  |
| Age mean SD | 49.7 (12.7) | 49.7 (17.4) |
| Sex |  |  |
| Female | 26.2% | 26.2% |
| Male | 73.8% | 73.8% |
| From long-term care home | 1.6% | 1.6% |
| Hospital site |  |  |
| 1 | 12.8% | 12.8% |
| 2 | 5.4% | 5.4% |
| 3 | 0% | 0% |
| 4 | 14.9% | 14.9% |
| 5 | 5.1% | 5.1% |
| 6 | 1.8% | 1.8% |
| 7 | 0.5% | 0.5% |
| 8 | 2.6% | 2.6% |
| 9 | 4.7% | 4.7% |
| 10 | 2.8% | 2.8% |
| 11 | 1.4% | 1.4% |
| 12 | 1.5% | 1.5% |
| 13 | 3.7% | 3.7% |
| 14 | 0.3% | 0.3% |
| 15 | 2.0% | 2.0% |
| 16 | 0% | 0% |
| 17 | 1.7% | 1.7% |
| 18 | 2.1% | 2.1% |
| 19 | 2.4% | 2.4% |
| 20 | 5.0% | 5.0% |
| 21 | 1.1% | 1.1% |
| 22 | 2.3% | 2.3% |
| 23 | 0.9% | 0.9% |
| 24 | 1.0% | 1.0% |
| 25 | 8.1% | 8.1% |
| 26 | 4.6% | 4.6% |
| 27 | 1.9% | 1.9% |
| 28 | 0.7% | 0.7% |
| 29 | 6.3% | 6.3% |
| 30 | 2.4% | 2.4% |
| 31 | 0% | 0% |
| Admission year |  |  |
| 2015 | 11.0% | 11.0% |
| 2016 | 11.1% | 11.1% |
| 2017 | 13.6% | 13.6% |
| 2018 | 17.2% | 17.2% |
| 2019 | 20.3% | 20.3% |
| 2020 | 12.7% | 12.7% |
| 2021 | 11.0% | 11.0% |
| 2022 | 3.2% | 3.2% |
| Admission season |  |  |
| Spring | 25.8% | 25.8% |
| Summer | 23.3% | 23.3% |
| Autumn | 23.3% | 23.3% |
| Winter | 27.6% | 27.6% |
| Comorbidities |  |  |
| Congestive heart failure | 1.6% | 1.6% |
| Chronic pulmonary disease | 7.1% | 7.1% |
| Connective tissue disease | 0% | 0% |
| Mild liver disease | 2.1% | 2.1% |
| Moderate to severe liver disease | 0.4% | 0.4% |
| Chronic kidney disease | 1.5% | 1.5% |
| Complicated diabetes mellitus | 1.3% | 1.3% |
| Malignancy | 2.7% | 2.7% |
| Metastatic cancer | 0.5% | 0.5% |
| Dementia | 0.2% | 0.2% |
| Hemiplegia | 0% | 0% |
| Cerebrovascular disease | 0.2% | 0.2% |

SD = standard deviation
